# Normalized emphysema scores on low dose CT: Validation as an imaging biomarker for mortality

**DOI:** 10.1371/journal.pone.0188902

**Published:** 2017-12-11

**Authors:** Leticia Gallardo-Estrella, Esther Pompe, Pim A. de Jong, Colin Jacobs, Eva M. van Rikxoort, Mathias Prokop, Clara I. Sánchez, Bram van Ginneken

**Affiliations:** 1 Department of Radiology and Nuclear Medicine, Radboud University Medical Center, Nijmegen, the Netherlands; 2 Department of Respiratory Medicine, University Medical Center Utrecht, Utrecht, the Netherlands; Universite de Bretagne Occidentale, FRANCE

## Abstract

The purpose of this study is to develop a computed tomography (CT) biomarker of emphysema that is robust across reconstruction settings, and evaluate its ability to predict mortality in patients at high risk for lung cancer. Data included baseline CT scans acquired between August 2002 and April 2004 from 1737 deceased subjects and 5740 surviving controls taken from the National Lung Screening Trial. Emphysema scores were computed in the original scans (origES) and after applying resampling, normalization and bullae analysis (normES). We compared the prognostic value of normES versus origES for lung cancer and all-cause mortality by computing the area under the receiver operator characteristic curve (AUC) and the net reclassification improvement (NRI) for follow-up times of 1–7 years. normES was a better predictor of mortality than origES. The 95% confidence intervals for the differences in AUC values indicated a significant difference for all-cause mortality for 2 through 6 years of follow-up, and for lung cancer mortality for 1 through 7 years of follow-up. 95% confidence intervals in NRI values showed a statistically significant improvement in classification for all-cause mortality for 2 through 7 years of follow-up, and for lung cancer mortality for 3 through 7 years of follow-up. Contrary to conventional emphysema score, our normalized emphysema score is a good predictor of all-cause and lung cancer mortality in settings where multiple CT scanners and protocols are used.

## Introduction

The presence of emphysema, visually assessed on CT images, has been shown to be a risk factor for lung cancer and overall mortality [1–4]. A known drawback of visual assessment is its subjectivity, which makes it susceptible to observer variability [5]. To overcome this limitation, computerized methods to objectively quantify emphysema have been developed. The most widely used measurement is the emphysema score (ES), defined as the percentage of lung voxels below a certain Hounsfield Unit (HU). ES has been previously associated with mortality in single center studies with a single imaging protocol [6, 7]. However, Martinez et al. [8] did not find any associations between ES and mortality risk in a study using data from multiple centers. Furthermore, Gierada et al. [9] showed only a weak association between ES and lung cancer in subjects derived from the multi-center National Lung Screening Trial (NLST).

A possible explanation for this discrepancy could be the heterogeneity of the reconstruction parameters used in multi-center studies, while single center studies all used the same imaging protocol. ES is known to vary with, among other factors, slice thickness and reconstruction kernel [10]. Hence, the variability introduced by the use of different reconstruction settings may obscure the association between ES and mortality.

Several methods have been suggested to overcome this problem. Blechschmidt et al. [11] proposed a morphology based method that classified bullae according to their size. The algorithm improved the results of emphysema quantification by ignoring isolated low attenuation voxels that were considered noise. Another way to reduce variability of ES across reconstruction kernels is to use a recently introduced normalization algorithm [12]. Results showed that normalized ES was independent of reconstruction settings and its correlation with lung function parameters was improved.

We present a normalized ES (normES) by applying resampling, normalization and bullae analysis prior to emphysema quantification. We hypothesize that normES is a better univariate predictor of mortality than the conventional emphysema score. Therefore, the purpose of this study is to develop a CT biomarker of emphysema that is robust across reconstruction settings, and evaluate its ability to predict mortality in patients at high risk for lung cancer, compared to conventional emphysema measurements.

## Materials and methods

### Ethics statement

Data was obtained from the National Lung Screening Trial (NLST) study, which obtained approval by an institutional review board at each screening center and all participants provided written informed consent in the study. Data received from the NLST study were de-identifyied/anonymized prior to access and analysis, therefore no identifiable information was used. For this reason, no institutional review board (IRB) approval was needed. Nonetheless, a waiver of approval was given by the Radboud University Medical Center IRB.

### Participants

This retrospective study used data from the CT arm of the NLST. The NLST is registered with clinical trial registration number NCT00047385 (https://clinicaltrials.gov/ct2/show/NCT00047385). Enrollment criteria and study design have been described previously [13]. The NLST study was a multicenter, randomized controlled trial in which participants enrolled at 33 centers in the United States underwent three annual screenings from August 2002 to April 2004 using either chest radiography or low-dose CT. The primary goal of the study was to compare the mortality rates in the low-dose CT arm with the mortality rates in the chest radiography arm.

Since the NLST study only allows to request the CT image data from a maximum of 6000 participants, we included all 1810 subjects that died during the trial (cases) and 4190 surviving controls (either censored or still alive at the end of the trial) randomly selected from the surviving group. With this study design, the odds ratio obtained will be approximately equal to the odds ratio in the full cohort, and it will also approximate the risk ratio in the complete cohort [14].

Only baseline scans (T0) were included in the present study, together with information about all-cause and lung cancer mortality outcomes obtained. Data used in this study is described in the project NLST-111 from the NCI Cancer Data Access System.

**Fig 1:**
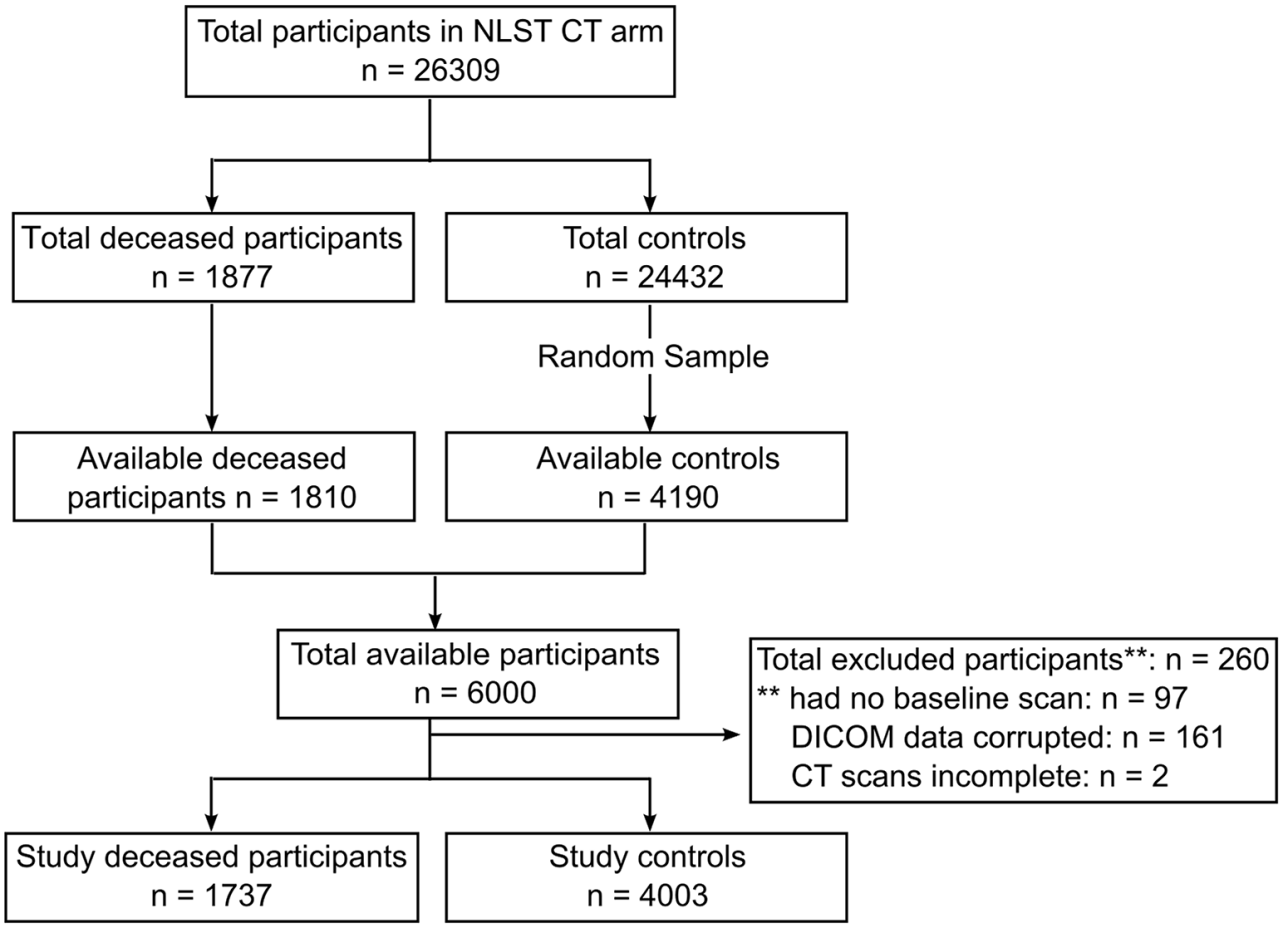
Selection of participants flow chart.

Of 6000 subjects, 260 patients were excluded for different reasons: they had no baseline CT scan (97), DICOM data was corrupted (161) or the CT images were not complete (2). This yielded a total of 5740 subjects (4003 alive, 1737 deceased) selected. Details of the flow of participants through the study are shown in Fig 1.

The study population was composed of 2174 (37.9%) women and 3566 (62.1%) men, with a median age of 61 years old (inter-quartile range, 57–65 years old), and 61 years old (inter-quartile range, 58–66 years old), respectively. The reconstruction settings for the baseline CT images are shown in S1 Table.

### Quantification of emphysema

Emphysema quantification was performed using CIRRUS Lung Quantification (Diagnostic Image Analysis Group, Nijmegen, The Netherlands; Fraunhofer MEVIS, Bremen, Germany). First, the lungs were automatically extracted using a segmentation algorithm based on region growing and morphological smoothing [15]. The extent of emphysema was then calculated using emphysema scores (ES), defined as the percentage of lung voxels with intensity values below −950 HU. Emphysema scores were computed in the original CT scans (origES) and in the images obtained after applying resampling to 3mm slice thickness, normalization and bullae analysis (normES). The goal of this processing is to reduce the variability in emphysema quantification produced by differences in slice thickness, reconstruction kernel and noise. The normalization reduces variability in ES as a result of varying reconstruction kernels by altering the appearance of CT scans to acquire similar characteristics as a reference kernel [12]. The bullae analysis algorithm detects air clusters inside the lungs and ignores those with a size lower than 5mm^2^ as they are assumed to be noise [11]. The parameters used in the aforementioned algorithms were calculated in the datasets described in [12] and [11], respectively. These datasets were completely independent from the NLST data. A detailed description of the algorithm to obtain normES can be found in S1 Appendix.

### Statistical analysis

To divide subjects in different categories based on their emphysema scores, we followed the same procedure as in the study of Johannessen et al. [7]. The degree of emphysema was divided in three categories as follows: a) low, for ES below the 60th percentile; b) medium, for ES between the 60th and 80th percentile; c) high, for ES higher than the 80th percentile.

Kaplan-Meier analyses were used to compute survival curves according to severity of emphysema. Pairwise log rank comparisons were conducted to determine which emphysema groups had different survival distributions. A Bonferroni correction was applied with statistical significance accepted at the p <0.0167 level. Since the death rate in our cohort is higher than in the full NLST study cohort, the alive sub-cohort was uniformly resampled to simulate the full alive cohort.

Time dependent receiver operating characteristic (ROC) curves and area under the ROC curve (AUC) were computed using non-parametric estimates for survival data [16–18] to evaluate the ability of origES and normES to predict all-cause and lung cancer mortality in a follow-up time of 1–7 years. Differences in AUCs were evaluated using bootstrap methods [19] with 6000 bootstrap samples.

To assess the added value of normES versus origES, we computed the continuous net classification improvement (NRI) for censored data [20, 21]. NRI quantifies the extent to which a biomarker assigns higher probabilities to individuals with outcome and lower probabilities to individuals without outcome compared to an initial biomarker.

Statistical analyses were performed using SPSS (version 23.0, IBM Corp., Armonk, NY) and R statistical package (version 3.2.1, R Foundation, Vienna, Austria) with packages “survMarkerTwoPhase” (version 1.1), “survival” (version 2.38.1) and “survIDINRI” (version 1.1).

## Results

Baseline demographic characteristics of study participants are shown in Table 1. The 60th and 80th percentiles for origES were 5.4% and 12.0%, respectively; and 0.5% and 1.9%, respectively for normES.

**Table 1:** Baseline characteristics by mortality status.

Characteristic	Alive	Deceased	Total
No. of subjects (%)	4003 (69.7)	1737 (30.3)	5740 (100)
No. of Male (%)	2350 (58.7)	1216 (70.0)	3566 (62.1)
No. of Female (%)	1653 (41.3)	521 (30.0)	2174 (37.9)
Median age (IQR)	60 (57–64)	63 (59–68)	61 (58–66)
Median pack-years (IQR)	47.3 (39.0–66.0)	55.5 (44.0–78.0)	50.0 (40.0–69.5)
No. of Current smoker (%)	1863 (46.5)	1032 (59.4)	2895 (49.6)
No. of Former smoker (%)	2140 (53.5)	705 (40.6)	2845 (50.4)
Median follow-up time in days (IQR)	2438 (2299–2556)	1503 (916–2000)	2344 (1941–2507)
No. of lung cancer deaths (%)		431 (24.8)	431 (100)

Data is presented as number (%), unless otherwise stated. IQR = inter-quartile range. Follow-up time is presented in days. Data was provided by the NCI Cancer Data Access System.

**Fig 2:**
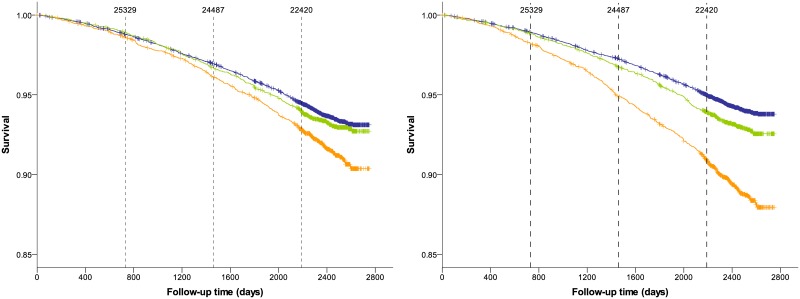
Kaplan-Meier survival estimates by emphysema categories for all-cause mortality when emphysema is quantified by computing origES (left) and normES (right). Blue, low emphysema category; green, medium emphysema category; orange, high emphysema category. Tick marks on the curves indicate censored data. Vertical dashed lines indicate time points at 730 days (2 years), 1460 days (4 years) and 2190 days (6 years). Number on top of the dashed vertical lines indicate the number of patients being followed up until the corresponding time point. Patients that are no longer followed up may be censored or deceased.

**Fig 3:**
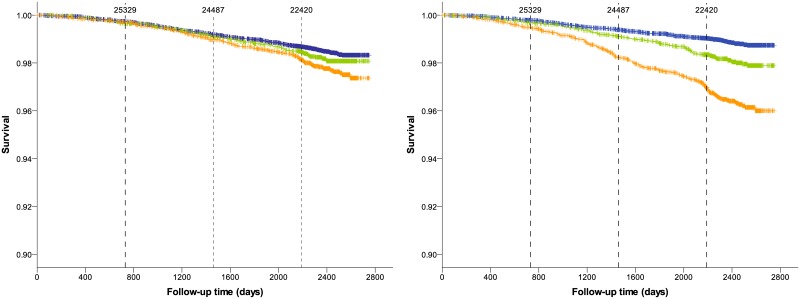
Kaplan-Meier survival estimates by emphysema categories for lung cancer mortality when emphysema is quantified by computing origES (left) and normES (right). Blue, low emphysema category; green, medium emphysema category; orange, high emphysema category. Tick marks on the curves indicate censored data. Vertical dashed lines indicate time points at 730 days (2 years), 1460 days (4 years) and 2190 days (6 years). Number on top of the dashed vertical lines indicate the number of patients being followed up until the corresponding time point. Patients that are no longer followed up may be censored or deceased.

Kaplan-Meier plots show survival estimates for the low, medium and high emphysema groups for all-cause and lung cancer mortality (Figs 2 and 3). We found that the differences in risk of all-cause and lung cancer mortality among emphysema categories became much more pronounced using normES instead of origES. Survival distributions were significantly different across all categories for all-cause mortality (p ≤ 0.003) and lung cancer mortality (p <0.001) when emphysema was quantified by normES. For origES and all-cause mortality, survival distributions were not statistically different between the low and medium emphysema categories (p = 0.272). For origES and lung cancer mortality, survival distributions were statistically different only between the low and high emphysema categories (p = 0.001).

NRI and ROC analysis results are shown in Table 2. This shows that normES is a superior predictor of all-cause mortality as compared to origES. The 95% confidence interval for the difference in AUCs indicates a significant difference between origES and normES for a follow-up time of 2–6 years. Furthermore, NRI values indicate a statistically significant improvement in classification for 2–7 years of follow-up when using normES.

**Table 2:** Discrimination of origES and normES, and net reclassification improvement for prediction of all-cause mortality.

Year	AUC origES (CI 95%)	AUC normES (CI 95%)	diffAUC (CI 95%)	NRI (CI 95%)
1	0.51 (0.46–0.57)	0.52 (0.46–0.57)	0.02 (−0.04–0.04)	6.9% (−1.7%–14.3%)
2	0.50 (0.47–0.54)	0.53 (0.50–0.57)	0.03 (0.00–0.05)	7.8% (2.0%–13.3%)
3	0.51 (0.48–0.54)	0.54 (0.51–0.56)	0.02 (0.00–0.04)	6.2% (3.0%–11.3%)
4	0.53 (0.51–0.55)	0.55 (0.53–0.58)	0.02 (0.01–0.04)	8.4% (4.6%–12.8%)
5	0.53 (0.51–0.55)	0.55 (0.54–0.57)	0.02 (0.01–0.04)	9.3% (5.5%–12.4%)
6	0.53 (0.51–0.55)	0.56 (0.54–0.57)	0.02 (0.01–0.04)	9.7% (7.05%–12.8%)
7	0.55 (0.53–0.58)	0.59 (0.55–0.60)	0.02 (−0.00–0.04)	10.1% (5.5%–14.5%)

AUC, area under the receiver operating curve; CI 95%, 95% confidence intervals; diffAUC, difference in AUC values between origES and normES; NRI, continuous net reclassification improvement.

A similar trend was observed for lung cancer mortality, as shown in Table 3. In this case, there was a significant difference between AUCS for origES and normES for follow-up times of 1–6 years and a significant improvement in classification for follow-up times of 3–7 years as indicated by the NRI values.

**Table 3:** Discrimination of origES and normES, and net reclassification improvement for prediction of lung cancer mortality.

Year	AUC origES (CI 95%)	AUC normES (CI 95%)	diffAUC (CI 95%)	NRI (CI 95%)
1	0.53 (0.40–0.66)	0.62 (0.50–0.73)	0.09 (0.03–0.15)	9.3% (−5.4%–29.1%)
2	0.52 (0.46–0.59)	0.59 (0.53–0.65)	0.07 (0.02–0.10)	9.2% (−0.8%–20.6%)
3	0.51 (0.46–0.56)	0.57 (0.53–0.62)	0.06 (0.03–0.10)	11.3% (1.2%–17.5%)
4	0.54 (0.50–0.58)	0.62 (0.58–0.65)	0.08 (0.05–0.10)	19.6% (11.5%–23.8%)
5	0.55 (0.52–0.58)	0.63 (0.60–0.66)	0.08 (0.05–0.10)	20.2% (14.9%–26.0%)
6	0.55 (0.52–0.58)	0.64 (0.61–0.67)	0.09 (0.06–0.11)	21.8% (16.8%–26.4%)
7	0.57 (0.54–0.60)	0.65 (0.62–0.68)	0.08 (0.05–0.11)	23.1% (18.0%–27.5%)

AUC, area under the receiver operating curve; CI 95%, 95% confidence intervals; diffAUC, difference in AUC values between origES and normES; NRI, continuous net reclassification improvement.

**Fig 4:**
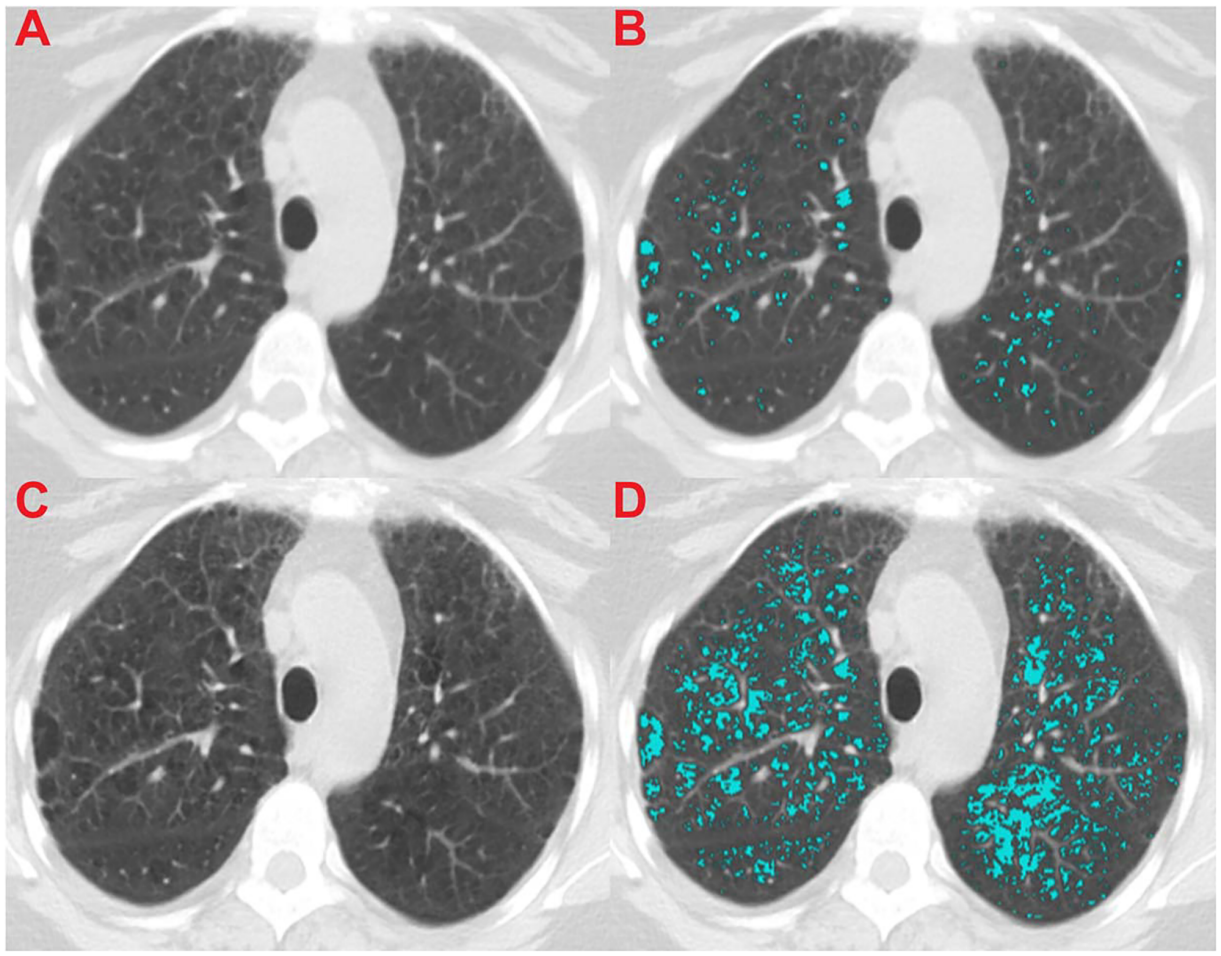
Illustration of a deceased subject that is categorized in the low emphysema group by origES (1.31%) and in the high emphysema group by normES (5.77%). The subject died after 1101 days. The CT image was acquired using a GE LightSpeed Pro 16 scanner and reconstructed with STANDARD kernel and 5mm slice thickness. (A) Shows the original CT section, (B) shows the original CT section with an emphysema overlay (origES), (C) shows the normalized CT section, and (D) shows the normalized CT section with a normalized emphysema overlay (normES).

**Fig 5:**
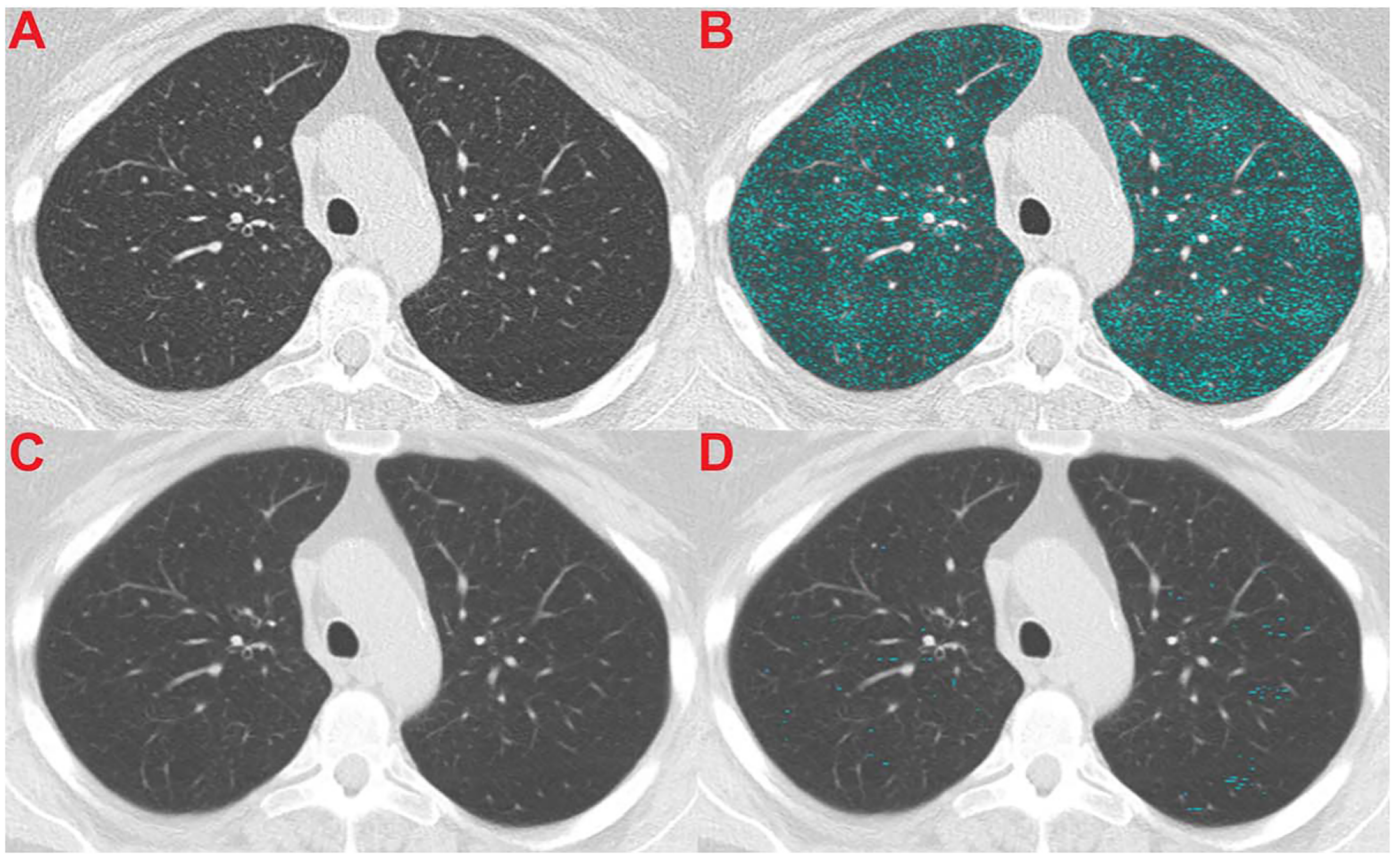
Illustration of an alive subject that is categorized in the high emphysema group by origES (32.44%) and in the low emphysema group by normES (0.45%). The subject was followed up for 2595 days. The CT image was acquired using a Siemens Sensation 16 scanner and reconstructed with B70f kernel and 2mm slice thickness. (A) Shows the original CT section, (B) shows the original CT section with an emphysema overlay (origES), (C) shows the normalized CT section, and (D) shows the normalized CT section with a normalized emphysema overlay (normES).

**Fig 6:**
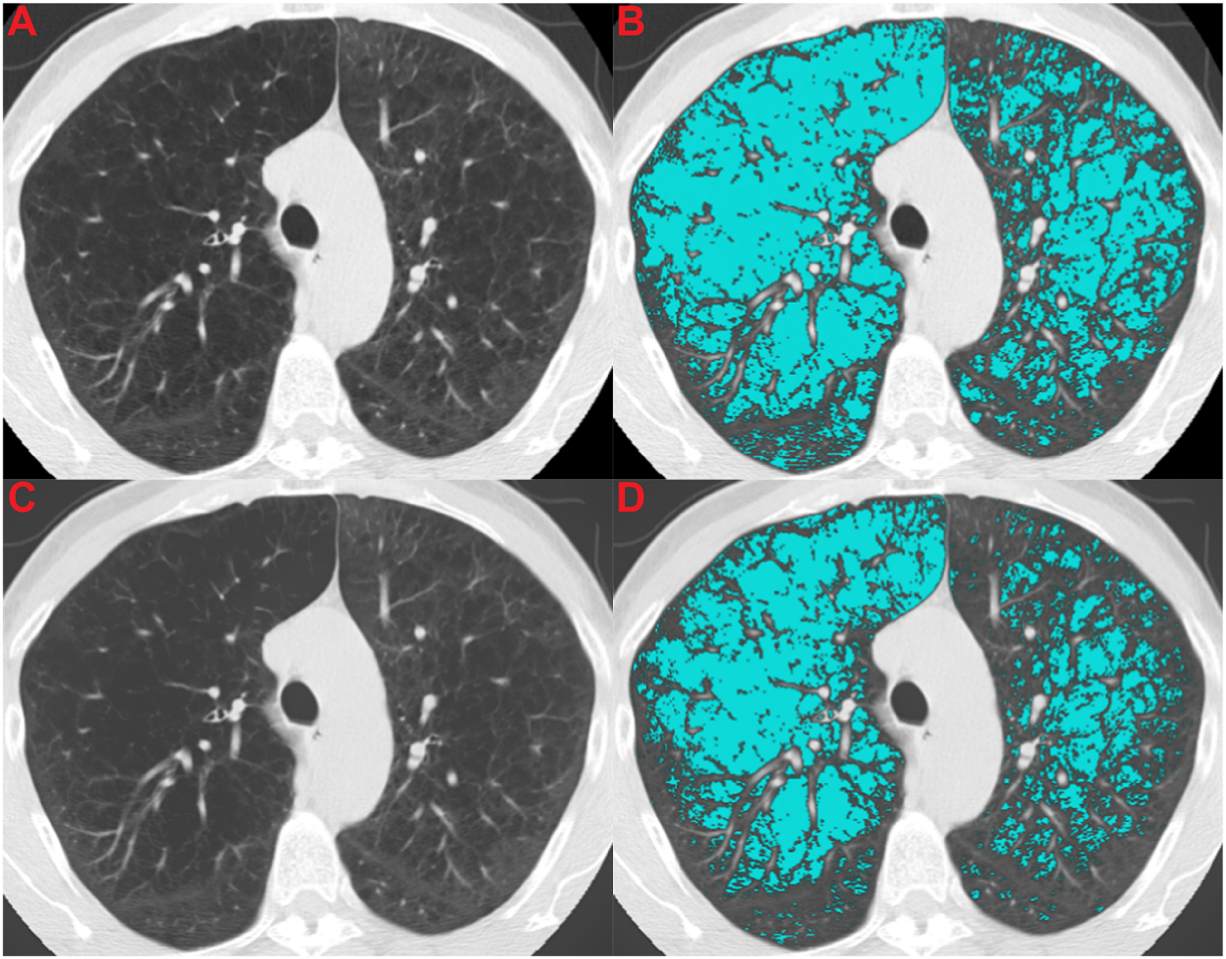
Illustration of a deceased subject that is categorized in the high emphysema group by origES (60.43%) and in the high emphysema group by normES (41.21%). The subject died after 2087 days. The CT image was acquired using a Toshiba Aquilion scanner and reconstructed with FC51 kernel and 2mm slice thickness. (A) Shows the original CT section, (B) shows the original CT section with an emphysema overlay (origES), (C) shows the normalized CT section, and (D) shows the normalized CT section with a normalized emphysema overlay (normES).

Figs 4–6 illustrate the effect of the normalization algorithm in subjects with different levels of emphysema.

## Discussion

This study shows that normES has a higher prognostic value than origES for all-cause and lung-cancer mortality in a large lung cancer screening cohort using CT data from multiple centers. Therefore, normES may be used as a robust biomarker for emphysema that could identify patients at increased risk of death and may benefit from early treatment or more frequent screening. To our knowledge, this is the first study that analyzes the relation between computerized emphysema quantification and mortality outcome in a large and heterogeneous database.

Several studies have analyzed the relationship between emphysema and mortality outcome before. Computerized emphysema scores have been found to be predictive of all-cause mortality in *α*_1_-antitrypsin deficiency patients [22] and in patients with various stages of COPD [6]. Johanessen et al. [7] showed that emphysema severity was associated with an increased all-cause mortality in a cohort of patients with and without COPD. Additionally, Zulueta et al. [4] also reported visual assessment of emphysema to be a significant predictor of lung cancer mortality in a lung cancer screening cohort.

Contrary to these findings, Martinez et al. [8] showed that computerized emphysema scores were not associated with mortality in a cohort of patients with severe emphysema, and Gierada et al. [9] showed only a weak association between emphysema quantification and lung cancer risk, using a smaller set of data from the NLST. We hypothesized that this discrepancy was due to differences in CT data induced by the acquisition protocol used: while the previously cited studies [4, 6, 7, 22] were single protocol, in the studies of Martinez et al [8] and Gierada et al. [9], the data was obtained from multiple centers with different imaging protocols. We also hypothesized that our normalization procedure based in a previously proposed algorithm to reduce variability in ES [12] should be able to correct for the confounding effect of CT acquisition protocols. This is illustrated in Figs 4–6, which show examples where normalized emphysema scores are in agreement with what can be visually assessed as emphysema, contrary to the standard emphysema scores that are too low when a soft kernel with thick sections is employed (Fig 4), and too high when a very sharp reconstruction kernel is used (Fig 5).

Our results are in accordance with both our hypotheses. Kaplan-Meier curves for all-cause mortality showed that there was an evident increase in risk of death already after 2 years of the baseline CT scan for patients with severe emphysema as measured by normES. However, for origES, there was no noticeable increase of risk until 4 years. This trend was even more obvious for lung cancer mortality, where the increase in risk of death was noticeable after 3 years for normES, whereas for origES it was only visible after 6 years. Furthermore, this improvement in risk reclassification for all-cause mortality was also observed for normES compared to origES for a follow-up time of 2 years and above.

Results on the association between emphysema and mortality are scarce, probably due to the fact that visual scoring of emphysema is time consuming and prone to inter-observer variability. Computerized emphysema scoring eliminates this variability but is still highly sensitive to CT reconstruction settings. This complicates the possibility of comparing data from different sources and thus impedes use in clinical routine, where variations in CT acquisition protocols and reconstruction kernels are inevitable. The presented normalization method overcomes these limitations and provides a robust emphysema biomarker that is easily computed automatically, can facilitate risk assessment and could be included in follow-up management strategies. Furthermore, normalized emphysema scores can be of value in lung cancer prediction models that not only consider nodule characteristics into account, but also include the presence of emphysema as a parameter [23].

Our study has some limitations. First, we analyzed the predictive value of emphysema quantification without taking into account other possible covariates. However, the goal of this study was not to create a complete prediction model for mortality, but to compare normalized emphysema scores to standard emphysema scores. Our results suggest that normES is a robust marker that may have an important prognostic value, especially in multi-center studies. We believe that it can improve the predictive ability of existing risk prediction models that includes the amount of emphysema as a variable. Second, we note that the AUC values obtained are not high, but this is to be expected as we are only using one marker, computed at baseline, to predict mortality over many years of follow-up. While further validation of normES in other lung cancer screening cohorts and as part of more elaborated lung cancer prediction models is needed, we believe that the results of this study can have relevant clinical implications in management and follow-up of patients in lung cancer screening programs.

## Conclusion

We have presented a robust CT imaging biomarker for emphysema that is associated with all-cause and lung cancer mortality in a high risk population regardless of the imaging protocol used. This biomarker can be used in risk prediction models, could improve follow-up management and might increase the cost-effectiveness of lung cancer screening programs.

## Supporting information

S1 Table Reconstruction parameters of the selected dataset. (PDF)

S1 Appendix Supplementary methods. Detailed description of the resampling, normalization and bullae analysis algorithms used in this work.
(PDF)

S1 Fig Illustration of the resampling method. (PDF)

S2 Fig Illustration of the separation of the original image into energy bands. (PDF)

S1 File origES and normES values for every subject in the dataset. (CSV)
