# Supplementary material for: Normalized emphysema scores on low dose CT: Validation as an imaging biomarker for mortality
Source: PLoS One. 2017 Dec 11;12(12):e0188902. doi: 10.1371/journal.pone.0188902 (PMC5724850; doi:10.1371/journal.pone.0188902)
Supplement: S1 Table — (PDF) [file pone.0188902.s001.pdf]

S1 Table. Reconstruction parameters of the selected dataset.

Manufacturer	Model	Kernel	Slice Thickness	KVp	mAs	# of scans
GE MEDICAL SYSTEMS	CT scan	STANDARD	2.5	120	60	8
GE MEDICAL SYSTEMS	CT scan	STANDARD	2.5	140	48	1
GE MEDICAL SYSTEMS	Discovery QX/i	STANDARD	2.5	120	40-72.5	43
GE MEDICAL SYSTEMS	HiSpeed QX/i	STANDARD	2.5	120	56-64	245
GE MEDICAL SYSTEMS	LightSpeed Plus	LUNG	2.5	120	80	4
GE MEDICAL SYSTEMS	LightSpeed Plus	STANDARD	2.5	120	30-102.5	328
GE MEDICAL SYSTEMS	LightSpeed Plus	STANDARD	2.5	140	40-80	190
GE MEDICAL SYSTEMS	LightSpeed Plus	STANDARD	3.75	120	73	1
GE MEDICAL SYSTEMS	LightSpeed Power	STANDARD	2.5	120	80	5
GE MEDICAL SYSTEMS	LightSpeed Pro 16	STANDARD	1.25	120	48	31
GE MEDICAL SYSTEMS	LightSpeed Pro 16	STANDARD	2.5	120	40-128	8
GE MEDICAL SYSTEMS	LightSpeed Pro 16	STANDARD	5	120	80	1
GE MEDICAL SYSTEMS	LightSpeed QX/i	STANDARD	2.5	120	48-80	168
GE MEDICAL SYSTEMS	LightSpeed QX/i	STANDARD	2.5	140	64	11
GE MEDICAL SYSTEMS	LightSpeed QX/i	BONE	2.5	120	40-56	6
GE MEDICAL SYSTEMS	LightSpeed QX/i	STANDARD	2.5	120	40-960	797
GE MEDICAL SYSTEMS	LightSpeed QX/i	STANDARD	2.5	140	40-80	244
GE MEDICAL SYSTEMS	LightSpeed Ultra	BONE	1.25	120	40	1
GE MEDICAL SYSTEMS	LightSpeed Ultra	BONE	2.5	120	25-75	21
GE MEDICAL SYSTEMS	LightSpeed Ultra	STANDARD	1.25	120	40-81	249
GE MEDICAL SYSTEMS	LightSpeed Ultra	STANDARD	1.25	140	60-120	3
GE MEDICAL SYSTEMS	LightSpeed Ultra	STANDARD	2.5	120	25-80	281
GE MEDICAL SYSTEMS	LightSpeed Ultra	STANDARD	2.5	140	55	1
GE MEDICAL SYSTEMS	LightSpeed16	BONE	2.5	120	30-60	29
GE MEDICAL SYSTEMS	LightSpeed16	BONE	2.5	140	60-80	8
GE MEDICAL SYSTEMS	LightSpeed16	STANDARD	1.25	120	40-80	59
GE MEDICAL SYSTEMS	LightSpeed16	STANDARD	1.25	140	70-200	3
GE MEDICAL SYSTEMS	LightSpeed16	STANDARD	2.5	120	36-80	280
GE MEDICAL SYSTEMS	LightSpeed16	STANDARD	2.5	140	40-80	69
Philips	Mx8000	A	3.2	120	79	1
Philips	Mx8000	B	1.3	120	60-105	9
Philips	Mx8000	B	3.2	120	40-87.5	36
Philips	Mx8000	C	1.3	120	45-150	128
Philips	Mx8000	C	2	120	140.25	2
Philips	Mx8000	C	3.2	120	37.5-122.25	323
Philips	Mx8000	C	3.2	140	70-99.75	6
Philips	Mx8000	D	3.2	120	39-60	7
Philips	Mx8000 IDT	B	2	120	63-85	2
Philips	Mx8000 IDT	C	2	120	45-75	48
SIEMENS	Sensation 10	B30f	2	120	30	1
SIEMENS	Sensation 16	B20f	2	120	45-60	3
SIEMENS	Sensation 16	B30f	1	120	75-125	143
SIEMENS	Sensation 16	B30f	2	120	25-90	428
SIEMENS	Sensation 16	B30f	2	140	70	1

SIEMENS	Sensation 16	B30f	5	120	90	1
SIEMENS	Sensation 16	B50f	1	120	37.5	1
SIEMENS	Sensation 16	B50f	2	120	45-60	12
SIEMENS	Sensation 16	B70f	2	120	49.5-54	9
SIEMENS	Sensation 16	B80f	1	120	125	1
SIEMENS	Sensation 4	B30f	2	120	40-80	88
SIEMENS	Sensation 4	B50f	2	120	40	8
SIEMENS	Volume Zoom	B30f	2	120	40-160	784
SIEMENS	Volume Zoom	B30f	2	140	35-165	49
SIEMENS	Volume Zoom	B30f	4	120	70	1
SIEMENS	Volume Zoom	B30f	5	120	60	2
SIEMENS	Volume Zoom	B50f	2	120	40-160	200
SIEMENS	Volume Zoom	B50f	2	140	40	1
TOSHIBA	Aquilion	FC02	1	120	40	2
TOSHIBA	Aquilion	FC02	2	120	40	2
TOSHIBA	Aquilion	FC51	2	100	50	1
TOSHIBA	Aquilion	FC51	2	120	40-80	343
TOSHIBA	Aquilion	FC51	2	135	75	1
TOSHIBA	Aquilion	FC53	2	120	40	1
