# Supplementary material for: Normalized emphysema scores on low dose CT: Validation as an imaging biomarker for mortality
Source: PLoS One. 2017 Dec 11;12(12):e0188902. doi: 10.1371/journal.pone.0188902 (PMC5724850; doi:10.1371/journal.pone.0188902)
Supplement: S1 Appendix — Detailed description of the resampling, normalization and bullae analysis algorithms used in this work. (PDF) [file pone.0188902.s002.pdf]

S1 Appendix. Supplementary methods

Resampling

As a first step, CT images were resampled in the z direction to non-overlapping sections of 3mm thickness to remove any variability due to different slice thicknesses. Essentially, the resampled image is the result of a weighted average of the original image. We can assume that every section in the resampled image represents a portion of the scanned volume. Similarly, that portion of scanned volume is represented in the original image by one or more sections. Therefore, we can compute the resampled section as a weighted average of the corresponding original sections. The weights were determined by the amount of scanned volume represented by the original section that is contained in the scanned volume represented by the resampled section. To illustrate this, an example is provided in Fig S1.

Normalization

After the images were resampled, a normalization algorithm [1] was applied to reduce variability in emphysema quantification due to varying reconstruction kernels. First, every image was decomposed into six frequency bands by subtracting the original image and its convolution with a Gaussian filter bank at six scales $\sigma = 0, 1, 2, 4, 8, 16$ voxels, as illustrated in Fig S2. The energy present in each frequency band, measured as the standard deviation of the intensity values in the lung, is then scaled to a reference value. This way, all normalized images have the same energy in every band. The reference values were obtained from an external dataset, not used in this study, composed of 183 scans reconstructed with a Siemens B31f kernel and 3mm slice thickness. The scan is then reconstructed by adding up all normalized frequency bands.

Bullae Analysis

After resampling and normalization, the emphysema mask was computed by selecting all those voxels in the normalized image that have an intensity value below -950 HU. This emphysema mask was subsequently post-processed using a bullae analysis algorithm [2] that classifies bullae depending on their size in sections of the scan. This is done by applying connected component labeling to the emphysema mask and sorting every component or bulla according to their size. Bullae with a size lower than 5mm2 are regarded as noise and therefore not considered emphysematous voxels; all bullae bigger 5mm2 than compose the voxels considered to compute the normalized emphysema score (normES).

References

1. Gallardo-Estrella L, Lynch DA, Prokop M, Stinson D, Zach J, Judy PF, et al. Normalizing computed tomography data reconstructed with different filter kernels: effect on emphysema quantification. Eur Radiol. 2016;26:478–486.

2. Blechschmidt RA, Werthschützky R, Lörcher U. Automated CT Image Evaluation of the Lung: A Morphology-Based Concept. IEEE Trans Med Imaging. 2001;20:434–442.
