# Supplementary material for: Normalized emphysema scores on low dose CT: Validation as an imaging biomarker for mortality
Source: PLoS One. 2017 Dec 11;12(12):e0188902. doi: 10.1371/journal.pone.0188902 (PMC5724850; doi:10.1371/journal.pone.0188902)

S1 Fig. Illustration of the resampling method.

In this example, we have an original image with 2mm slice thickness and 1.5mm voxel spacing to be resampled to 3mm slice thickness and 3mm voxel spacing. The figure illustrates the computation of the section 0 (dark blue) of the resampled image. The light blue region and the braces with the blue dotted lines indicate the information from the scanned volume that should be contained in the resampled section 0 (dark blue), considering that we have a (resampled) slice thickness of 3mm. To compute the resampled section, we use the sections of the original image that contain information of this region. Again, in the original image, the brackets with the dotted lines indicate which region of the scanned volume is contained in each section (color coded). So, looking at these braces, we see that sections 0 (red), 1 (green), 2 (purple) in the original scan contain information of the region we are interested in. Therefore, we could average these sections to obtain our resampled one. However, it is important to note that the information contained in original sections 0 (red) and 1 (green) is inside our region to compute, but original section 2 (purple) is not completely inside our region to compute. Therefore, we use a weighted average, in which section 2 (purple) would have a lower weight, proportional to the amount of information that is inside our region to compute. This process is repeated for every section in the resampled image.

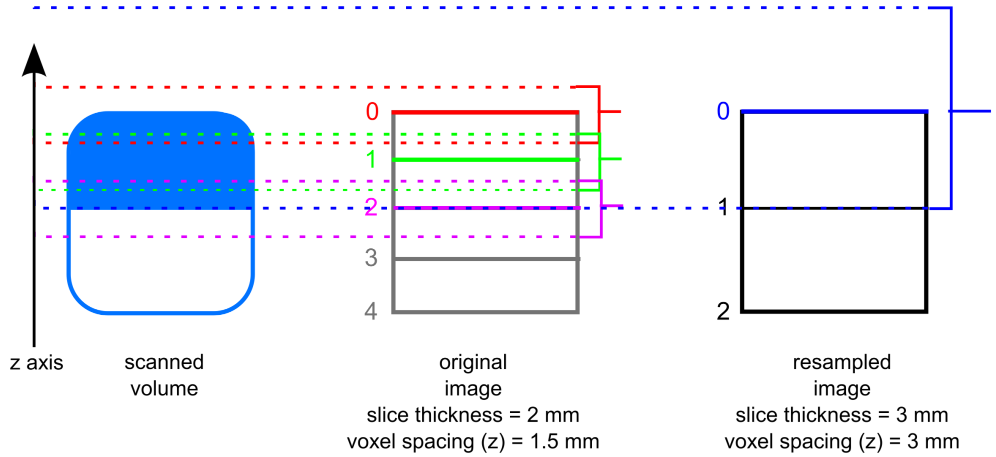

Supplement: S1 Fig — (PDF) [file pone.0188902.s003.pdf]
