# Supplementary material for: Normalized emphysema scores on low dose CT: Validation as an imaging biomarker for mortality
Source: PLoS One. 2017 Dec 11;12(12):e0188902. doi: 10.1371/journal.pone.0188902 (PMC5724850; doi:10.1371/journal.pone.0188902)

# S2 Fig. Illustration of the separation of frequency bands.

This figure illustrates the process to separate the original image into frequency bands, performed by subtracting the original image ($I_{orig}$) and the original image convoluted with a Gaussian at different scales ($G_i$). $F_i$ denotes the frequency bands, with $F_1$ being the highest frequency and $F_6$ the lowest.

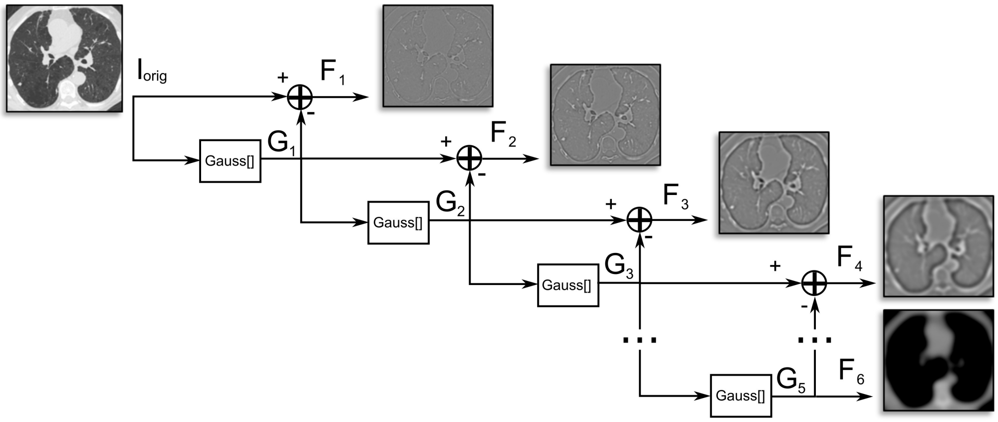

Supplement: S2 Fig — (PDF) [file pone.0188902.s004.pdf]
